# Rheumatoid arthritis prevalence in Quebec

**DOI:** 10.1186/1756-0500-7-937

**Published:** 2014-12-19

**Authors:** Sasha Bernatsky, Alaa Dekis, Marie Hudson, Christian A Pineau, Gilles Boire, Paul R Fortin, Louis Bessette, Sonia Jean, Ann L Chetaille, Patrick Belisle, Louise Bergeron, Debbie Ehrmann Feldman, Lawrence Joseph

**Affiliations:** Division of Clinical Epidemiology, McGill University Health Centre, 687 Pine Avenue West, V-Building, V2.09, Montreal, QC H3A 1A1 Canada; Division of Rheumatology, McGill University Health Centre, Montreal, Quebec Canada; Jewish General Hospital and Lady David Research Institute, Montreal, Quebec Canada; Department of Medicine, University of Sherbrooke, Sherbrooke, Quebec Canada; Faculty of Medicine, Laval University, Quebec City, Quebec Canada; Rheumatology Department, Centre Hospitalier Universitaire de Québec (CHUQ) Research Centre, Quebec City, Quebec Canada; Chronic Disease Surveillance Division, National Institute of Public Health of Québec, Quebec City, Quebec Canada; Canadian Arthritis Patient Alliance, Ile Perrot, Quebec Canada; Université de Montréal, École de réadaptation, Montreal, Quebec Canada

**Keywords:** Arthritis, Epidemiology, Rheumatoid Arthritis

## Abstract

**Background:**

To estimate rheumatoid arthritis (RA) prevalence in Quebec using administrative health data, comparing across regions.

**Methods:**

Cases of RA were ascertained from physician billing and hospitalization data, 1992–2008. We used three case definitions: 1) ≥ 2 billing diagnoses, submitted by any physician, ≥ 2 months apart, but within 2 years; 2) ≥ 1 diagnosis, by a rheumatologist; 3) ≥1 hospitalization diagnosis (all based on ICD-9 code 714, and ICD-10 code M05). We combined data across these three case definitions, using Bayesian hierarchical latent class models to estimate RA prevalence, adjusting for the imperfect sensitivity and specificity of the data. We compared urban versus rural regions.

**Results:**

Using our case definitions and no adjustment for error, we defined 75,760 cases for an over-all RA prevalence of 9.9 per thousand residents. After adjusting for the imperfect sensitivity and specificity of our case definition algorithms, we estimated Quebec RA prevalence at 5.6 per 1000 females and 4.1 per 1000 males. The adjusted RA prevalence estimates for older females were the highest for any demographic group (9.9 cases per 1,000), and were similar in rural and urban regions. In younger males and females, and in older males, RA prevalence estimates were lower in rural versus urban areas.

**Conclusions:**

Without adjustment for error inherent in administrative databases, RA prevalence in Quebec was approximately 1%, while adjusted estimates are approximately half that. The lower prevalence in rural areas, seen for most demographic groups, may suggest either true regional variations in RA risk, or under-ascertainment of cases in rural Quebec.

## Background

There is growing interest in developing tools and methods for the surveillance of chronic rheumatic diseases, using existing resources such as administrative health databases. Comparing disease prevalence across certain regions might be of particular interest; for example, for historic and geographic reasons, individuals in some rural regions of Quebec have been somewhat isolated from other parts of the province. This has many potential effects; one may be differences in genetic make-up, and other may be variations in access to care. Differences in access to care might mean that the sensitivity and specificity of administrative data-based case definitions may vary across rural versus urban areas, and even within rural areas, from one sub-region to another.

Our objective was to estimate the prevalence of rheumatoid arthritis (RA) in Quebec based on administrative health data, and to determine if RA prevalence estimates were any different in urban versus rural regions. We also performed exploratory sub-analyses in two regions which have been particularly isolated geographically. Since administrative data rely on medical contact in order to ascertain cases, the RA prevalence estimates in these very isolated areas (where access to care is presumably lower) might be different from the rest of rural Quebec. On the other hand, very isolated regions generally tend to have reduced genetic variation [1–3] which theoretically could alter RA risk (compared to the rest of Quebec), since genetic susceptibility is a risk factor (albeit a complex one) for RA [4]. We sought to provide new data regarding the estimates of RA prevalence across regions in Quebec, including these very isolated regions. The methodological approach chosen in this paper offers a means of dealing with the imperfect nature of administrative data, as will be seen.

## Methods

We used hospitalization and physician billing data for all of Quebec (approximately 7.6 million residents), across 1992–2008, to estimate the prevalence of existing RA cases in 2008. The data include hospitalization discharge diagnoses (a primary diagnosis and 15 non-primary diagnoses per hospitalization, abstracted by medical records clerks) and physician visit billing claim diagnostic codes (a single diagnostic code is allowed per visit). All, diagnoses are provided as International Classification of Diseases (ICD-9 and ICD-10) codes (RA being represented by ICD-9 code 714, and ICD-10 code M05). In the hospitalization data, we defined a potential RA case as any hospitalization that included RA as a primary or non-primary discharge diagnosis. In the billing data, potential cases were required to have 2 or more RA diagnostic codes (by any physician) at least 2 months apart but within a 2-year span. A second alternative algorithm defined a potential case on the basis of at least one RA diagnostic code during a visit to a rheumatologist. Cases aged <18 were not excluded. Deceased residents are recorded by Quebec vital statistics and the information provided with the linked administrative data.

Once potential RA cases in Quebec were identified by one or more of these case definitions, we used our previously developed Bayesian latent class hierarchical models to generate prevalence estimates from these data, adjusting for the imperfect sensitivity and specificity of these sources [5, 6]. This approach does not rely on a gold standard (which is unavailable in these administrative data). Instead, multiple case definitions each provide some information about the case status of subjects. This allows the disease status for each subject to be estimated probabilistically, and the sum of these probabilities provides the number of estimated cases. The sensitivity and specificity estimates produced from this model are relative to the true disease status of subjects, which is not known and is thus a ‘latent’ variable. This allows simultaneous estimation of disease prevalence, as well as the sensitivity and specificity of each case definition [7]. Bayesian methods use probability distributions to reflect uncertainty about parameters in a model [8]. One begins with a ‘prior distribution’ which may be ‘uninformative’ (where the results will thus be ‘informed’ mainly by the data) or ‘informative’ (which describe likely starting values for a parameter of interest). Our Bayesian latent class model accounted for possible conditional dependence between our ascertainment methods, that is, the possibility that the ascertainment methods are dependent even conditional on the (unknown) true case status. This model is non-identifiable without informative prior input on at least two parameters. Based on previous work on rheumatic disease case ascertainment using administrative data [9], we expected the specificities of all methods to be very high. Therefore, for our primary analyses we set informative prior distributions for the specificities of our three case ascertainment approaches. This prior distribution corresponds to specificities of 98% (potential values ranging from approximately 96–100%). This choice was based on our early work in systemic autoimmune rheumatic disease [10] and is supported by data from a recent validation study of administrative data which suggest that algorithms that incorporate RA billing data have a specificity of 99-100% [11].

We constructed 95% credible intervals (95% CrI) representing the values between which there is a 95% probability of containing the parameter of interest, given the data and the prior information used. All programming was carried out using WinBUGS software (MRC Biostatistics Unit, University of Cambridge, Cambridge, UK).

Since RA prevalence is known to vary according to age and sex, we derived age and sex-specific estimates, based on the demographic data found in the administrative data sources. Information on region of residence was based on the forward sortation area information for subjects (that is, the first three digits of an individual’s Canadian postal code) which is available in the health care administrative database. We calculated period prevalence based on the estimated number of cases ascertained over 1992–2008, using census statistics to determine the appropriate population denominator and the group-specific prevalence per 100,000 residents. The hierarchical nature of our Bayesian latent class model allowed for differences in sensitivity of the different three case definitions in capturing RA within populations defined by sex, age group, and residence.

Two sub-regions of rural Quebec for which we produced specific estimates of RA prevalence included Les Îles de la Madeleine (in the Gulf of Saint Lawrence), and the Saguenay/Lac-St-Jean census metropolitan area, in the northern-east part of Quebec. Les Îles de la Madeleine form a small archipelago in the Gulf of Saint Lawrence with a land area of 205.53 square kilometres, forming a census division and a three-digit postal code region (G4T). There are 8 major islands, all but one inhabited, but only two municipalities, Les Îles-de-la-Madeleine (2006 census pop. 12,560), and Grosse-Île (pop. 531). The Saguenay-Lac-St-Jean region is located in the northern-eastern part of Quebec, and the vast majority of its population is of French-Canadian descent, with a unique and relatively limited genetic pool. The region has remained relatively geographically isolated and has a current population size of approximately 285,000 individuals.

Rheumatology access in these two regions has been problematic for some time. From 1990 to 2000, rheumatologists from Quebec City travelled 2–3 days a month to Saguenay/Lac-St-Jean to see patients. After this, patients from the Saguenay/Lac-St-Jean region travelled to Quebec City to see rheumatologists. A single rheumatologist began to work in Saguenay/Lac St-Jean only after the end date of our study interval. Similarly, one rheumatologist from Quebec City travels to Iles de la Madeleine three times a year, to conduct one week of clinic, but this is a more recent practice. For the period of our observation interval, most RA patients from Îles de la Madeleine were followed by local generalist physicians. Our study was approved by the McGill University research ethics board, without requirement for consent, as we did not have direct access to patients, or nominal information.

## Results

Using our case definitions and no adjustment for error, we defined 75,760 individuals who met one or more of the RA definitions at least once from 1992 onward, and remained alive up to 2008. This number translates into unadjusted estimates of an over-all RA prevalence of 9.9 per thousand residents. With these case definitions and no adjustment for error, we defined 139 RA cases in 13,110 individuals, (representing an over-all prevalence of 10.6/1000) in Iles de la Madeleine. In Saguenay/Lac-St-Jean, we defined 1,094 RA cases in 138,671 residents (7.9/1000). In the rest of Quebec, this algorithm defined 74,527 cases, within a population of 7,464,237 (10.0/1000).

Our Bayesian hierarchical latent class model estimates, adjusting for the imperfect sensitivity and specificity of our case definition algorithms, suggested a Quebec RA prevalence of 4.83 cases per 1,000 persons (95% CrI 4.80, 4.86). As expected, the prevalence estimate was higher in females at 5.57 per 1000 (95% CrI 5.54, 5.60) versus males (4.06 per 1000; 95% CrI 4.01, 4.11).

The results from our Bayesian model, stratified by residence and age/sex groups, are indicated in Table 1. RA prevalence estimates for older females were identical in rural and urban regions. However, RA prevalence estimates tended to be lower in rural versus urban regions for all other age-sex groups. Generally, estimates of RA prevalence were considerably higher in older versus younger individuals. This is to be expected, since RA is a chronic disease with onset occurring in working force age and older. In younger individuals, RA prevalence estimates were clearly higher for females versus males, which again reflects the known female-predominance of RA during young adulthood, with a diminishing difference in the female: male ratio in older age groups. The highest prevalence was in older urban residents, where RA prevalence was about 9.9 cases per 1,000 (essentially 1%).

**Table 1:** **Rheumatoid arthritis in rural and urban Quebec: Period prevalence (1992–2008) estimates (per 1,000 residents) according to age and sex categories, based on latent class model regression**

Region	Age and sex group	Prevalence	95% CrI*
		[per 1000]	
Rural	Female 45+	9.95	9.94, 9.95
	Female < 45	1.53	1.44, 1.62
	Male 45+	7.45	7.20, 7.70
	Male < 45	0.47	0.43, 0.52
Urban	Female 45+	9.95	9.95, 9.95
	Female < 45	2.05	1.97, 2.13
	Male 45+	9.94	9.90, 9.95
	Male < 45	0.73	0.68, 0.78

*Bayesian 95% credible intervals the values between which there is a 95% probability of containing the parameter of interest, given the data and the prior information used.

**Figure 1:**
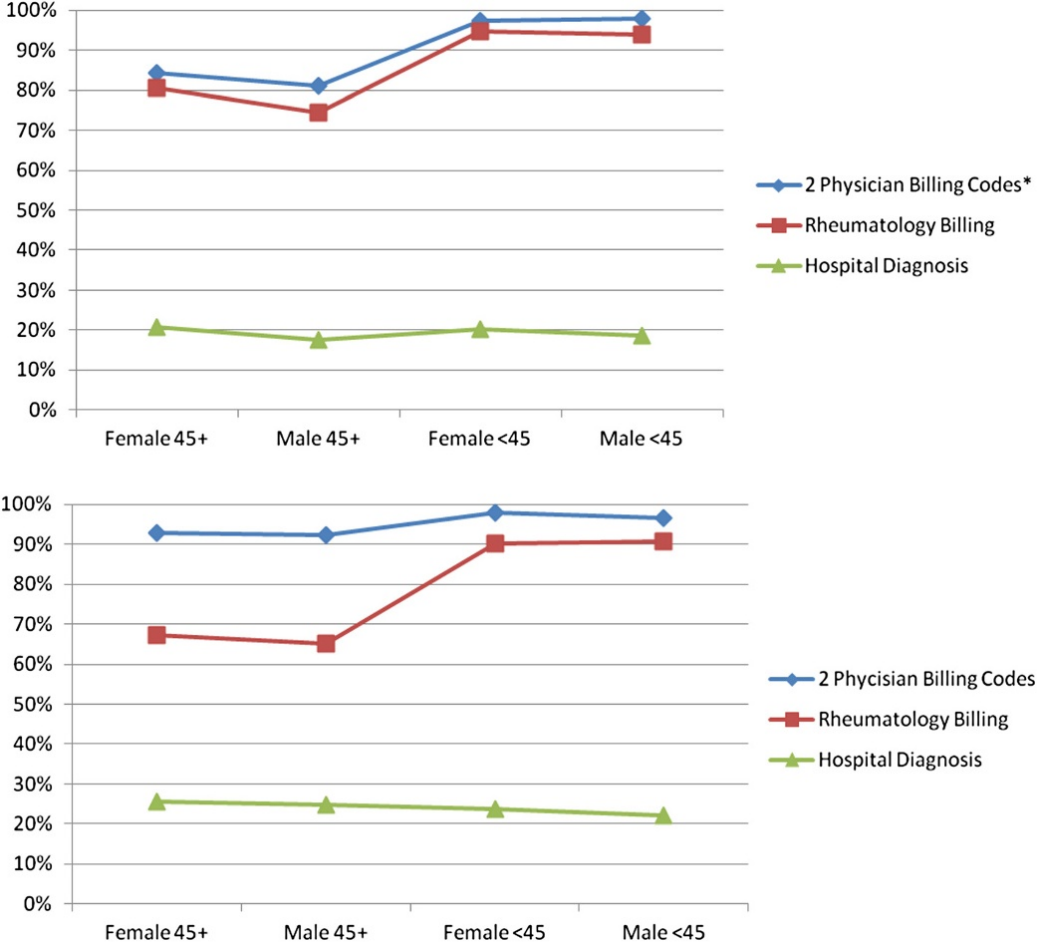
**Sensitivity estimates for the different case definitions based on International Classification of Diseases (ICD) codes for rheumatoid arthritis (RA), within administrative data: Variations in urban versus rural residence, according to age and sex groups.** *2 billing codes by any physician for RA, at least 8 weeks apart but within 2 years. One or more hospital discharge diagnoses for RA, including, both primary and non-primary discharge diagnoses (up to 15). At least one billing code for RA by a rheumatologist.

Table 2 displays the prevalence estimates for age and sex specific strata in the regions of both Îles de la Madeleine and Saguenay, compared to over-all Quebec rural rates. In older individuals, the prevalence estimates for both males and females were similar to the over-all rural RA prevalence estimates for these demographic groups. In addition, within the bounds of the credible intervals, the estimates for males and for older females were similar (though not identical) in the regions of both Saguenay/Lac-St-Jean and Iles de la Madeleine, compared to the over-all rural RA prevalence estimates for these demographic groups. However, in both sub-regions of Saguenay/Lac-St-Jean and Iles de la Madeleine, RA prevalence for younger women was estimated as slightly higher, than the over-all Quebec rural estimate for this demographic group.Figure 1 shows the sensitivity estimates for the different case definitions, according to age and sex categories, for rural versus urban residence. In both regions, the algorithm based on two physician billing codes was estimated to be the most sensitive of the three case definitions, across all age and sex groups. Both case definitions that used billing data tended to be more sensitive in younger (versus older residents). This was more pronounced for the algorithm based on one or more rheumatology billing code for RA, particularly in rural areas. This figure is interesting in that it could suggest that individuals older than 45 are less likely to be seen by rheumatologists in rural, than in urban, areas. As might be expected, the sensitivity of hospitalization diagnoses for RA case ascertainment was low (since most RA patients would not be hospitalized).

**Table 2:** **Rheumatoid arthritis in over-all rural Quebec and in two rural sub-regions: Period prevalence (1992–2008) estimates (per 1,000 residents) according to age and sex categories, based on latent class model regression: Contrasting rural Quebec results to two rural sub-regions**

Region	Age and sex group	Prevalence	95%	CrI**
		[per 1000]		
Rural Quebec*	Female 45+	9.95	9.94	9.95
	Female < 45 y/o	1.53	1.44	1.62
	Male 45+	7.45	7.20	7.70
	Male < 45 y/o	0.47	0.43	0.52
**Iles de la Madeleine**	Female 45+	9.65	9.00	9.94
	Female < 45 y/o	4.00	2.00	7.22
	Male 45+	7.17	4.24	9.71
	Male < 45 y/o	0.37	0.00	2.10
**Saguenay**	Female45+	9.90	9.68	9.95
	Female < 45 y/o	2.30	1.77	2.99
	Male 45+	5.50	4.45	6.80
	Male < 45 y/o	0.60	0.38	0.99

*Estimate for rural Quebec does not include the two sub-regions.

**Bayesian 95% credible intervals the values between which there is a 95% probability of containing the parameter of interest, given the data and the prior information used.

## Discussion

Although a vast majority of the land area in Quebec is rural, twenty percent of Quebec residents live in rural areas. Of these twenty percent, 7% live on farms and the remaining 93% live in small towns. Many rural areas, including the Lac St. Jean and Iles des Madeline regions, are predominantly French-speaking, although in some regions, particularly northern regions, first-nations groups are represented. The population of Quebec’s rural communities as a whole includes slightly more children (below age 15) and seniors (age 65 and over) than Quebec’s urban areas. Employment conditions, incomes, and education levels are all far better in urban areas. The further the distance from the large centres, the more these economic characteristics generally deteriorate [12].

Without adjustment for error inherent in administrative databases, RA prevalence in Quebec was approximately 1%, while adjusted estimates are approximately half that. In comparison, using administrative databases but without adjustment for imperfect case definition, age and sex standardized RA prevalence estimates in Ontario from 1996–2010 have been estimated at 0.5%-0.9% [13].

Our results suggested that prevalence estimates tended to be lower in rural versus urban areas, for most demographic groups (although the prevalence estimates for older females were identical between rural and urban regions). We found that RA prevalence estimates in two rural regions (Îles de la Madeleine and Saguenay-Lac-St-Jean) were similar to the over-all estimates for rural Quebec. This was in contrast to our suspicion that these areas, being more remote than the rest of rural Quebec, might have less access to medical care, and subsequently lower RA prevalence estimates based on administrative data. The one difference was observed in younger women from these two sub-regions, who had higher RA prevalence estimates than in the rest of Quebec. Our results therefore do not preclude the possibility of some genetic predisposition (or other risk factor) driving RA prevalence in these areas.

Most data from the developed world suggest RA prevalence estimates between 0.5% and 1%; our estimate of over-all RA prevalence in Quebec is at the lower end of this spectrum, which could be related to various factors. First, published estimates vary greatly in terms of methods; additionally, it has been suggested that there is a trend for a slight decrease in RA prevalence over the past several decades. A study from Rochester, Minnesota showed a prevalence of clinically confirmed RA in 1985 to be 1.07% (95% confidence interval [95% CI] 0.94–1.20) among adults aged >35 years of age; using the same methods, the estimate of RA prevalence fell to 0.85% in 1995 (95% CI 0.75–0.95) [14]. More recent data suggest an RA prevalence in the US of about 0.6% [15], which is similar to our own adjusted estimate.

Our estimates of the sensitivity of the different case definitions suggest that without the case definition capturing two physician visits for RA, prevalence would indeed be under-estimated, particularly in rural areas. That is, at least in Quebec, case definitions based on rheumatologist billing alone, will likely miss proportionally more RA in rural, than in urban, areas. This could lead to falsely low estimates of RA prevalence in rural areas. An approach similar to ours, which combines more than one case definition, and adjusts for imperfect specificity and sensitivity, may then be more useful than reliance on a single case definition.

Although rheumatology care is indeed scarce in the Saguenay/Lac St. Jean and Iles-de-la Madeleine regions, there are issues in other parts of the province as well; in fact there are no rheumatologists located in the large expanses of Cote Nord (which encompasses much of the northern shore of the Saint Lawrence River estuary and the Gulf of Saint Lawrence) and Nord du Quebec (which covers most of the Labrador Peninsula) [16]. Based on Canadian Medical Association (CMA) statistics for the distribution of rheumatologists, Quebec has been slightly above the Canadian average of 1.01 rheumatologists per 100,000 residents [17]. This observation is limited by the fact that CMA statistics for the number of rheumatologists in Canada may not actually reflect active practice, accounting for the number of part-time versus full-time rheumatologists or their academic versus clinical practice.

Potential limitations of our study relate to the fact that RA cases were not clinically validated, and that the data reflect only persons seeking medical care, who are given an RA diagnosis on billing or hospitalization data. Moreover, physicians can only provide one billing diagnosis per visit, and physicians not infrequently assign diagnostic codes even prior to confirming a diagnosis [18].

The requirement of ‘2 or more physicians billing diagnoses, at least 2 months apart and within a 2 year span’ reflects modelled after an early administrative data-based algorithm first presented by Maclean et al. [19] and modified for use by others [20, 21]. This approach has also been used by other chronic disease researcher (e.g. for diabetes research and surveillance) and is based on the knowledge that a single billing code lacks specificity (in RA, one validation study suggested a specificity of only 62.5%) [11, 22]. Our approach disregards codes that are close in time (less than 2 months) since presumably a significant proportion of those represent follow-up visits where the diagnosis is being ruled out. [18] At the same time, it is presumed that there should be no more than a 2 year period between codes, since a longer period might make it unlikely that the subject truly has the disease in question.

In fact, the Public Health Agency of Canada includes similar case definition in their surveillance definitions, and across all provinces, it functions well in terms of providing reasonable and stable estimates of disease prevalence [23]. Of course, there have been some studies using 3 or more billing diagnoses (Shipton et al.) [24] and a recent validation study in Canada confirmed preservation of sensitivity (83%) and accuracy (81-82%). [11] The validation study showed moreover that increasing the stringency of the algorithm, such as by limiting cases to those only with rheumatology billing code diagnoses, actually decreased accuracy (78%) [11].

We used Bayesian latent class regression to combine information from different case definitions, and based on our earlier experience with this approach, and the use of multiple sources of administrative data for rheumatic case identification, we assigned high prior values for specificity of the billing code algorithms. Though there have been some recent validation studies which found specificities that were somewhat lower [22] for physician-billing algorithms, these were ***not*** population-based studies, but rheumatology-clinic based. That is, the ‘gold standard’ was a rheumatologist diagnosis and the validation samples were ***only*** selected from rheumatology offices, not the general population. The one available study that was population-based, using administrative data case definitions similar to ours (with case validation done via chart review of unselected family medicine records, not rheumatology clinics) found that the specificity of physician billing case definition algorithms were 97% [11].

RA is more common among women than men, particularly during reproductive years. In our analyses, the estimated prevalence among men age 45 and older was only slightly lower than that of women in this age group. In fact, similar trends have been noted in an epidemiologic study in Ontario, [13] representing an established tendency for the female-to-male ratio in RA, to diminish in older age. Since RA prevalence also increases steeply with age, in the categories of age 45 and older, a very considerable number of the cases in this subgroup are likely to be seniors (between age 65 and 85), a demographic where males and females have more similar RA risk, than in younger individuals [25]. Our Bayesian approach did allow us to estimate sensitivity differences according to sex, and as indicated in our figure (which importantly, also considers potential differences in sensitivity across sex and urban–rural groups), our estimates of sensitivity for the algorithms did not appear to be particularly affected by sex. As mentioned in the introduction, previous work on rheumatic disease case ascertainment using administrative data had estimated that the specificities of all methods as very high, and these did not differ by sex [9].

Finally it should be noted that our intent was to provide an RA prevalence estimate based on the whole population of interest, without focussing on, or excluding, pediatric-onset RA or juvenile idiopathic arthritis (JIA). Cases aged <18 represent only a small component of the RA billing codes; in pediatrics JIA includes some entities that one might consider more in keeping with the concept of ‘juvenile rheumatoid arthritis’ and others where the adult counterpart (more or less) includes what, in adults, is considered “sero-negative arthritis”, not RA. That being said, we have actually been working with the PHAC and members of the Canadian Alliance of Pediatric Rheumatology Investigators (CAPRI) to develop an alternative approach to the JIA case definition, which incorporate a wider range of ICD codes (i.e. to include juvenile-onset seronegative arthopathies).

## Conclusions

On one hand, our results are of considerable interest, in that the effects of poor access to medical care in more remote areas may not be as dramatic as has been feared. This is somewhat re-assuring, given recent efforts on the part of the Public Health Agency of Canada to use administrative data for chronic disease surveillance [26]. On the other hand, the results do not rule out poor access to rheumatology care across ***all*** of rural Quebec. We cannot under-estimate the potential public health problem resulting from this. Thus, caution must still be exercised in the interpretation of prevalence estimates based on these data, and further attempts should be made to optimize the methodological approaches using administrative data for rheumatic disease research and surveillance [27].

## Abbreviations

RA: Rheumatoid arthritis
ICD-9: International Classification of Diseases, 9^th^ revision
ICD-10: International Classification of Diseases, 10^th^ revision
Pop: Population
95% CrI: 95% credible intervals
95% CI: 95% confidence interval
CMA: Canadian Medical Association.
